# Sensitivity of nonlinear photoionization to resonance substructure in collective excitation

**DOI:** 10.1038/ncomms7799

**Published:** 2015-04-09

**Authors:** T. Mazza, A. Karamatskou, M. Ilchen, S. Bakhtiarzadeh, A. J. Rafipoor, P. O'Keeffe, T. J. Kelly, N. Walsh, J. T. Costello, M. Meyer, R. Santra

**Affiliations:** 1 European XFEL GmbH, Albert-Einstein-Ring 19, 22761 Hamburg, Germany; 2 Center for Free-Electron Laser Science, DESY, Notkestrasse 85, 22607 Hamburg, Germany; 3 Department of Physics, University of Hamburg, Jungiusstrasse 9, 20355 Hamburg, Germany; 4 Stanford PULSE Institute, SLAC National Accelerator Laboratory, 2575 Sand Hill Road, Menlo Park, California 94025, USA; 5 CNR Istituto di Struttura della Materia, CP10, I-00016 Monterotondo Scalo, Italy; 6 School of Physical Sciences and NCPST, Dublin City University, Dublin 9, Ireland

## Abstract

Collective behaviour is a characteristic feature in many-body systems, important for developments in fields such as magnetism, superconductivity, photonics and electronics. Recently, there has been increasing interest in the optically nonlinear response of collective excitations. Here we demonstrate how the nonlinear interaction of a many-body system with intense XUV radiation can be used as an effective probe for characterizing otherwise unresolved features of its collective response. Resonant photoionization of atomic xenon was chosen as a case study. The excellent agreement between experiment and theory strongly supports the prediction that two distinct poles underlie the giant dipole resonance. Our results pave the way towards a deeper understanding of collective behaviour in atoms, molecules and solid-state systems using nonlinear spectroscopic techniques enabled by modern short-wavelength light sources.

## Abstract (web summary)

Electrons in atoms exhibit many-body collective behaviours that can be studied by highbrightness X-rays from FELs. Here, the authors examine two-photon above threshold ionization of xenon and find that nonlinearities in the response uncover that more than one state underpins the 4*d* giant resonance.

## Graphical abstract

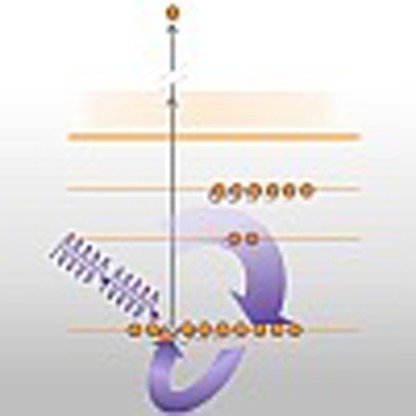

Significant advancements in photonics^1^,^2^,^3^, especially in electric field enhancement^4^,^5^ and harmonic generation^6^, have been mostly triggered by the recent development in tailoring materials on the nanometre scale exploiting their resonant collective response to radiation^7^. To optimize the coupling between the nanostructure and the electromagnetic field, a detailed understanding of the underlying resonant response is essential. To this end, atomic samples provide a valuable benchmark for understanding more complex systems, easing meaningful systematic investigations.

An illustrative example of a many-body system showing collective electronic behaviour is atomic xenon^8^; its resonating character under extreme ultraviolet (XUV) radiation, known as the 4*d* giant dipole resonance, is interpreted as the collective response of many electrons to an external weak-field perturbation^9^,^10^. The recent advent of high-brilliance light sources such as XUV and X-ray free-electron lasers (FELs) has opened a door to XUV and X-ray studies beyond the linear regime. Exploiting this new high-intensity technology renders it possible to investigate the collective response mechanisms of many-body systems through their nonlinear interaction with short-wavelength radiation. As shown here, this provides the possibility of unveiling substructures in the spectrum of collective excitations that cannot be resolved with linear spectroscopy.

The case of xenon ionization under the unprecedented conditions at FELs has been the subject of several investigations^11^,^12^,^13^,^14^,^15^,^16^,^17^, which have stimulated speculations about the influence of collective effects on the process of multiple ionization^12^,^14^; furthermore, a high harmonic generation experiment on xenon^18^ evidenced the impact of the 4*d* giant resonance on a nonlinear optical process^19^,^20^. Yet, all these observations can be well understood, as far as collectiveness is concerned, in terms of the 1-photon absorption cross-sections of the various charge states of xenon^13^,^17^,^21^, that is, in terms of the spectral characteristics of its linear response.

Employing nonlinear electron spectroscopy, namely through the study of xenon 2-photon ionization, we demonstrate here that the nonlinear process unveils otherwise unresolved aspects of the collective behaviour of the system. Due to the photon energy selected, the 2-photon process occurs through the giant resonance as an intermediate step (Fig. 1). We show, however, that a model assuming a single intermediate state cannot describe our results. Instead, the resonance feature in the predicted energy dependence of the 2-photon process and its shape strongly suggest that more than one resonance state underlie the giant resonance^22^; although these states are unresolved in the linear ionization of xenon, 2-photon ionization turns out to be a sensitive process for their observation.

## Results

### Experiment and theory approach to nonlinear photoionization

**Figure 1:**
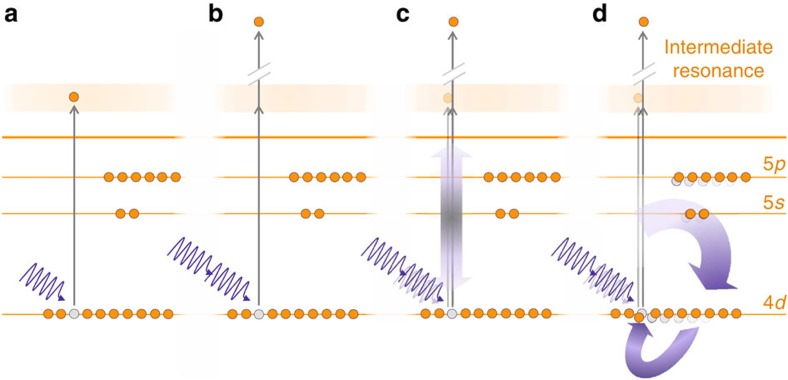
Schematic representation of the ionization processes and associated models. (**a**) 1-photon ionization process; (**b**) 2-photon ionization process; (**c**) 1- and 2-photon processes according to the reduced model, only including interaction of the emitted electron with the hole from which it is excited; (**d**) 1- and 2-photon processes according to the full model, accounting for electron–hole interaction in all channels open to ionization.

Our findings are made possible by the combination of electron spectroscopy, which allows the disentanglement of photoemission processes from different orders of interaction, with first-principles calculations. We measured the relative yields of 1-photon and 2-photon ionization of the 4*d* shell of xenon (Fig. 1a,b) by electron spectroscopy and compare them with numerical solutions of the many-electron Schrödinger equation for atomic xenon in the presence of an external XUV laser field. Our theoretical model captures many-body processes beyond linear response theory, allowing the selective inclusion of those electronic correlation effects that are responsible for collectiveness. For a system characterized by collective behaviour, the wavefunction is given by a coherent superposition of particle–hole states^23^, due to the strong particle–hole interaction. We compare the experimental results with the full model, which describes the collective response of the system by accounting for the electron–hole interaction in all channels open to ionization (Fig. 1d), and a reduced model, which confines this interaction to the hole from which the electron was excited (Fig. 1c).

### Electron spectroscopy of 1- and 2-photon ionization

Our experiments were performed at the BL2 beamline of FLASH^24^, the Free-electron LASer in Hamburg, Germany. FEL pulses at photon energies of 105 and 140 eV were focused down to a few microns in front of the aperture of a magnetic bottle electron spectrometer. The spectrometer was used to measure the kinetic energy (KE) of the electrons produced by 1-photon and 2-photon absorption processes in an effusive jet of xenon atoms (see Methods).

**Figure 2:**
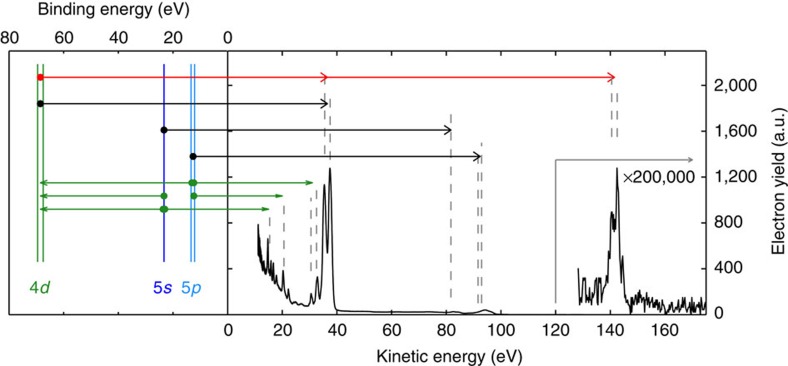
Electronic level scheme and emission spectrum. Electron spectrum from XUV-ionized xenon atoms, recorded at *hν*=105 eV with a FEL irradiance of (6±2) × 10^12^ W cm^−2^, is shown along with the energy level scheme for the xenon orbitals involved in the ionization processes. The spectrum includes features coming from electron emission caused by different processes represented by arrows: 1-photon direct emission (black), Auger emission (green) and 2-photon direct emission (red). In the low-KE region (KE <50 eV) the spectrum is dominated by the contribution from the 4*d* (1-photon) photoemission and by the subsequent Auger decays involving the 5*s* and the 5*p* shells. The small features at KE between 80 and 100 eV arise from the 1-photon photoemission from 5*s* and 5*p* shells. The high-energy feature is assigned to the 2-photon photoemission from the 4*d* shell.

Electron spectra (Fig. 2) were collected under different intensity conditions. The spectra include features caused by 1-photon direct emission from the 5*p*, 5*s* and 4*d* shells as well as from NOO Auger decay^25^. At higher kinetic energies, the 2-photon ionization from the 4*d* shell is observed in a spectral feature that resembles in shape the 4*d* (1-photon) emission lines and is separated from them by exactly the energy of one photon.

**Figure 3:**
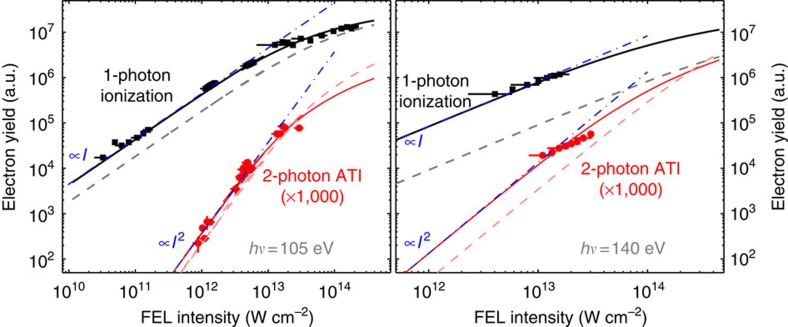
Intensity dependence of 1-photon and 2-photon photoemission yields. Experimental electron yields as a function of FEL intensity are extracted integrating the electron spectra recorded at 105 eV (left pane)/140 eV (right pane) photon energy in the 33–39 eV/68–74 eV (1-photon 4*d*, plotted in black squares) and 136–146 eV/206–216 eV (2-photon 4*d*, plotted in red circles) KE ranges. The 105 eV photon energy, 1-photon 4*d* electron yield is extracted by subtracting the partially overlapping Auger electron spectrum; the contribution of the latter is estimated from the literature^25^ using the two Auger peaks at 30 and 32 eV KE as a normalization reference. The vertical error bars in the experimental 2-photon yields represent the statistical error. Horizontal error bars include uncertainty in the pulse energy, focal spot size and pulse duration measurements. Thin blue dash–dotted lines with slopes indicated in blue are drawn to highlight the linear and quadratic dependence of the 1-photon and 2-photon yields, respectively, in the low-intensity region. The experimental yields are compared with theoretical yields based on the full (solid lines) and the reduced (dashed lines) models for both 1-photon (bold lines) and 2-photon (thin lines) yields.

The relative yields from the 4*d* 1- and 2-photon ionization processes are obtained by integrating the spectra over the corresponding kinetic energies regions (105 eV, 1-photon: 33–39 eV; 140 eV, 1-photon: 68–74 eV; 105 eV, 2-photon: 136–146 eV; 140 eV, 2-photon: 206–216 eV; see caption of Fig. 3) and are shown as a function of the FEL intensity in Fig. 3. At low intensities (*I*<10^13^ W cm^−2^), the 1- and 2-photon ionization yields show a linear and quadratic dependence, respectively. This confirms, on the basis of perturbation theory, the nature of the ionization processes. At higher intensities, the depletion of the neutral target induced by the enhanced 1-photon ionization leads to a pronounced saturation effect.

### Comparison between experimental and theoretical results

The experimental yields are compared with the results of calculations (Fig. 3) performed for the full and the reduced models, respectively. The theoretical yields are obtained from the numerical solutions of rate equations (see Methods). The comparison between experimental points and rate equation solutions employs a single normalization factor for all data sets (1-photon and 2-photon yields at 105 and 140 eV, respectively).

This comparison clearly shows that the full model reproduces the intensity dependence of the experimental yields, whereas the reduced model fails to do so. This means that the inclusion of Coulomb coupling between all possible electron–hole states, which is responsible for the collective electronic response of the system, is an essential ingredient for the correct description of the 2-photon ionization process. The very good agreement is evident in the ratio between the 1-photon and 2-photon ionization yields at both photon energies over the whole intensity range as well as in the onset of the saturation due to neutral target depletion.

## Discussion

**Figure 4:**
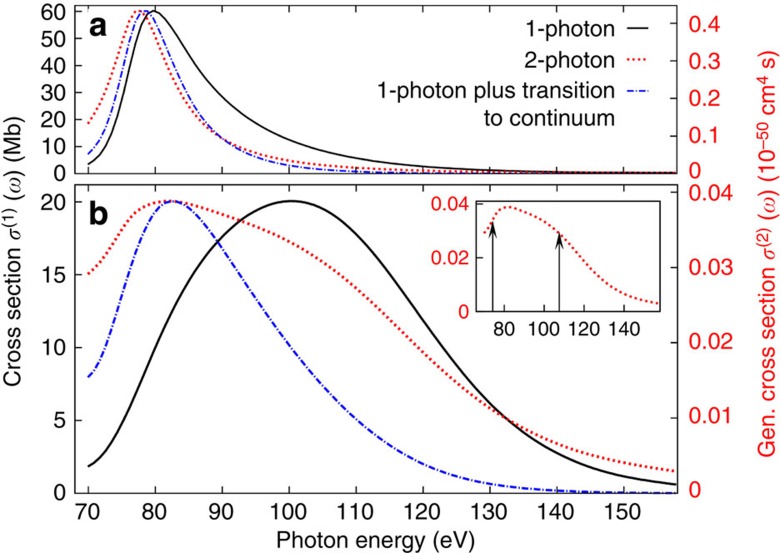
Photon energy dependence of the calculated cross-sections. Photon energy dependence of the 1-photon (solid black line) and 2-photon (dotted red line) cross-sections calculated with the reduced model (**a**) and the full model (**b**). The scales on the left and right axes are chosen such that the maxima of the curves appear at the same height as the 1-photon cross-section peak. The dash–dotted blue lines represent the result for the 2-photon cross-section within the two-step model with one single intermediate resonance state. In the case of the reduced model, this approach captures the main features of the 2-photon cross-section, while for the full model it breaks down. The inset shows the full model 2-photon cross-section with two arrows indicating the energy position of the two underlying resonances calculated within the TDCIS model.

Having validated our full model by the comparison with experimental yields at two photon energies, we investigate the influence of collective effects on the 1- and 2-photon ionization cross-section over a wide photon energy range (Fig. 4). For the 1-photon cross-section, the broadening is due to the well-known broadening and blue shift of the giant resonance caused by the inclusion of coupling among different electron–hole states^9^, which is reproduced by our calculations (Fig. 4).

As a mostly unexpected and counter-intuitive result, the full model predicts a much broader 2-photon cross-section curve than for the 1-photon case. Considering the 2-photon cross-section within perturbation theory, the 2-photon cross-section curve produced by the reduced model can be qualitatively understood (Fig. 4a) in terms of a sequential process involving a single intermediate state, where the 2-photon cross-section (red-dotted curve) factorizes into two 1-photon cross-sections (one photon for exciting the giant resonance from the ground state (solid black curve), and the other photon for the transition from the resonance to the final state, which is modelled by a *E*^−13/2^ energy dependence (see Supplementary Discussion)). According to this two-step picture with a single intermediate state, one expects a narrower 2-photon peak that is shifted to lower energy (dash–dotted blue curve), since the 1-photon cross-section for exciting an electron from the intermediate state into the continuum decreases monotonically with increasing energy. This model captures qualitatively the behaviour of the 2-photon cross-section in the reduced model case. In contrast, for the full model (Fig. 4b), the picture of a sequential process involving a single intermediate state does not hold: surprisingly, the 2-photon cross-section curve is much broader than the 1-photon cross-section curve and exhibits a knee-type structure. This substructure, which emerges in the nonlinear process, manifests the existence of more than one resonance state underlying the giant resonance^22^. These states give rise to interference terms resulting in a broadening of the 2-photon absorption cross-section curve (see Supplementary Discussion). Indeed, the experimental results cannot be explained, simultaneously at 105 and 140 eV, by the two-step picture with a single intermediate state (dash–dotted blue curve). In particular, at 140 eV the cross-section measured experimentally is ∼12 times larger than predicted by the single intermediate state model, while at 105 eV it is larger by a factor of 2.2. Further analysis within the time-dependent configuration interaction singles scheme (TDCIS) reveals two underlying resonance states^26^, which are indicated by arrows in the inset of Fig. 4b. The resonance positions are consistent with the substructure visible in the 2-photon cross-section. Here for the first time, the agreement of a theoretical model with experimental results beyond the linear regime legitimizes the prediction of two resonances underlying the giant resonance^22^.

Summarizing, we have shown that the nonlinear response of an electronic system to intense XUV radiation can be used to unveil information about the collective behaviour in many-body systems. The theoretical xenon 2-photon cross-section exhibits a knee-type structure that is not visible in the 1-photon cross-section. The present study demonstrates, employing xenon as a model system, how the nonlinear interaction regime can be utilized to investigate collective electronic behaviour. This stands only at the beginning of the way towards a deeper understanding of the collective response of many-body systems.

## Methods

### FEL beam transport and characteristics

The self-amplified spontaneous emission FEL pulses had a duration of about 80±20 fs and up to 40 μJ (at 105 eV) and 15 μJ (at 140 eV) energy per pulse. The bandwidth was about 1% at both photon energies. The FEL pulses were focused onto the sample by means of MoB_4_C multilayer mirrors in a back-reflecting geometry to produce a tight focusing of 5±1 μm full width at half maximum. The mirrors have a reflection bandwidth of ∼1 eV with peak reflectivity of ∼40% (at 105 eV) and ∼20% (at 140 eV) centred at the respective photon energy, thus enabling in addition a very effective filtering (>4 orders of magnitude) of any possible higher harmonic contamination (estimated <0.3%) that might be present in the FEL beam^24^. FEL irradiance was tuned using a gas attenuator system and moving the focusing mirror along the beam direction in order to vary the beam cross-section within the interaction zone. The attenuator was used to control the energy per pulse delivered into the interaction region thereby providing a fine tuning of the intensity over a restricted range (∼1 order of magnitude). In addition, by varying the beam cross-section from the minimum value of 5 μm up to ∼190 μm, the intensity was altered over more than 4 orders of magnitude. The photon beam parameters were monitored online during the experiments. A calibrated gas monitor detector provided the energy of the FEL pulses on a single-shot basis^24^. A charge-coupled device camera was used to record the single-shot FEL spectra from a variable line spacing grating spectrometer installed along the beam transport. The spectral information was used to normalize the beam intensity to the multilayer mirror reflection curve.

### Electron spectroscopy for determining experimental yields

Electron spectra of 1-photon and 2-photon ionization of xenon were measured by means of a magnetic bottle electron spectrometer (MBES)^27^. Technically, since the photon energies exceed the binding energy of the orbitals considered, the observed 2-photon process is above-threshold ionization.

The acceptance volume of the MBES, limited by the magnetic field lines of the system, had a size of ∼0.5 mm in the plane perpendicular to the spectrometer axis. The MBES enables 4π acceptance of the solid angle with an energy resolution for the detected electrons of 2%. By means of a retardation stage, it was possible to increase the resolution of the spectral features down to the FEL bandwidth limit.

The 1-photon and 2-photon signals were collected for different FEL intensities under different MBES settings as well as different conditions for the sample density. 2-Photon electrons were collected under higher sample density conditions and applying a retarding field at the entrance of the MBES rejecting slow electrons, to avoid detector saturation induced by the 1-photon signal. The intensity-independent normalization factors defining the relative yields (sample density, transmission of the analyser and detector gain) are calibrated by comparing the experimental and theoretical results obtained for the 1-photon and 2-photon ionization from the 3*p* orbital of argon, which is a much less complex system exhibiting negligible correlation effects, thereby providing a robust calibration reference.

The experimental intensity domains are not identical for the the 1-photon and the 2-photon yields, collected in subsequent measurements, because of the consistent varying of the self-amplified spontaneous emission FEL intensity during the shifts. For the 105-eV case, where electron yields are more severely affected by saturation effects at high intensities, the experiment was performed under different focusing conditions to allow the investigation over a broader intensity range. The experimental yields are extracted by integrating the FEL intensity-resolved electron spectra in the KE regions mentioned in the Results section, corresponding to the binding energy ranges from 66 to 72 eV and from 64 to 74 eV for the 1-photon and the 2-photon signals, respectively.

### Data acquisition

Electron energy spectra were acquired by feeding the signal from the detector collection anode into a Lecroy WavePro 725Zi-A digital oscilloscope (8 bit, 10 GSPS, 2.5 GHz bandwidth) triggered by a transistor–transistor logic signal synchronized with the FEL pulse. The DAQ server was controlled by a Labview-based data acquisition (DAQ) client enabling the collection of single-shot spectra and their sorting according to the intensity and the spectral distribution of the FEL. Intensity-resolved electron energy spectra can be extracted in two different ways depending on the energy region examined. Low-KE spectra, produced by single-photon processes, result from the analogue current signal collected by the detector anode. The 2-photon direct ionization features, with yields that are some orders of magnitude lower than the 1-photon features, result from the collection of only a few electrons per FEL shot by the detector. Their signal is time discriminated by software, and the histogram of their arrival time is taken in counting mode and suitably normalized, resulting in a virtually background-free electron spectrum. This approach enables the extension of the dynamic range well beyond the limitation given by the digitizer.

### First-principles calculation of cross-sections

Our model is based on the TDCIS^28^. In this nonperturbative approach the full N-electron Schrödinger equation is solved numerically


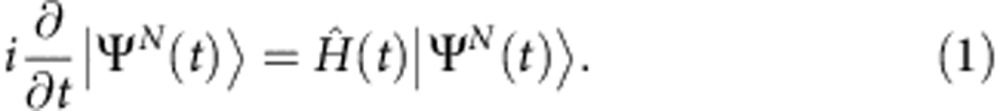


The wavefunction is expanded in the one-particle–one-hole basis:


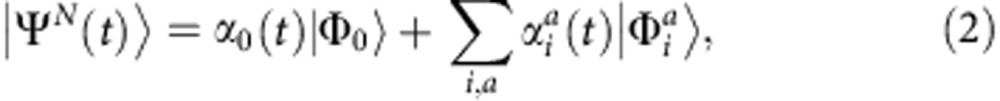


where the index *i* denotes an initially occupied orbital, *a* stands for an unoccupied orbital and |Φ_0_〉 symbolizes the Hartree–Fock ground state. The cross-sections for 1- and 2-photon absorption are calculated via the population in the corresponding hole channels, which are distinguishable due to the different angular momenta of the ejected electron. The level of our calculations does not include any ground-state correlations. Within TDCIS it is possible to include and distinguish certain electronic correlation effects that are mediated by Coulomb interaction. In particular, for the description of a collective response, the system cannot be described by a single particle–hole state, but rather a superposition of particle–hole states is needed. The full model includes the coupling among the holes in the 4*d*, 5*s* and 5*p* orbitals and the electron. The corresponding Coulomb matrix elements 
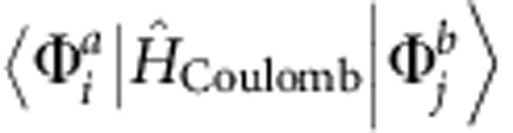
 are included for all different index pairs within the space of active orbitals. In this way, superpositions of particle–hole states, that is, collective states, may be described. In contrast, in the case of the reduced model the elements with *i*≠*j* are set to zero, which results in the description of coupling only with the very 4*d* orbital from which the electron was ionized.

### Rate equations for theoretical yield calculation

The theoretical yields are obtained from the numerical solution of equations (3, 4, 5, 6, 7) valid for the electron yield from the neutral target (population *N*_0_).


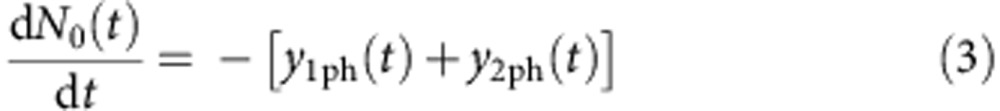



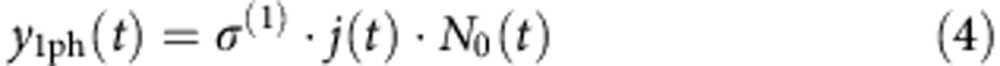



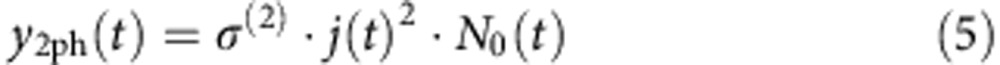



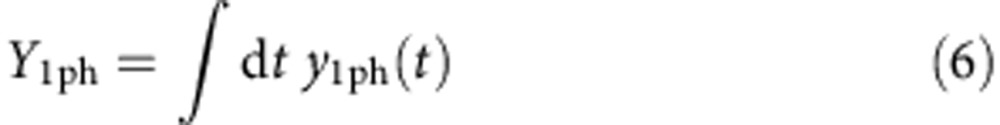



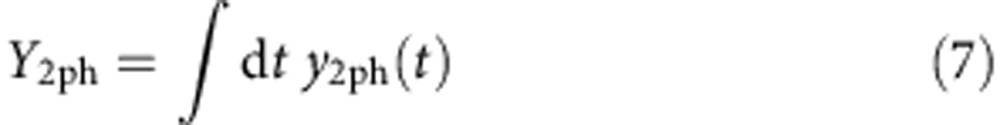


Rate equations are solved assuming a Gaussian pulse with 80 fs (full width at half maximum) duration. *σ*^(1)^ (1-photon) and *σ*^(2)^ (2-photon) ionization cross-sections entering equations (4) and (5) are obtained for the full and the reduced model as described above. The rate equation solutions (*Y*_1ph_, *Y*_2ph_) are calculated over a very broad range (9 orders of magnitude) of laser intensities and the results are numerically integrated over the volume of acceptance of the electron analyser in order to account for the spatial distribution of the FEL fluence.

## Author contributions

The experiment was conceived by M.M., coordinated by T.M. and M.M. and performed by T.M., M.I., S.B., A.J.R., P.O.K., N.W., T.J.K., J.T.C. and M.M.; A.K. and R.S. performed the numerical calculations and elaborated the theoretical model; T.M. and A.K. analysed and combined the results from experiment and theory; the manuscript was prepared by T.M., A.K., M.M. and R.S. with contributions from all the authors.

## Additional information

**How to cite this article:** Mazza, T. *et al.* Sensitivity of nonlinear photoionization to resonance substructure in collective excitation. *Nat. Commun.* 6:6799 doi: 10.1038/ncomms7799 (2015).

## Supplementary Material

Supplementary Information Supplementary Figure 1, Supplementary Discussion and Supplementary References
