# Supplementary material for: Sensitivity of nonlinear photoionization to resonance substructure in collective excitation
Source: Nat Commun. 2015 Apr 9;6:6799. doi: 10.1038/ncomms7799 (PMC4403373; doi:10.1038/ncomms7799)
Supplement: Supplementary Information — Supplementary Figure 1, Supplementary Discussion and Supplementary References [file ncomms7799-s1.pdf]

Supplementary Information

Supplementary Figure

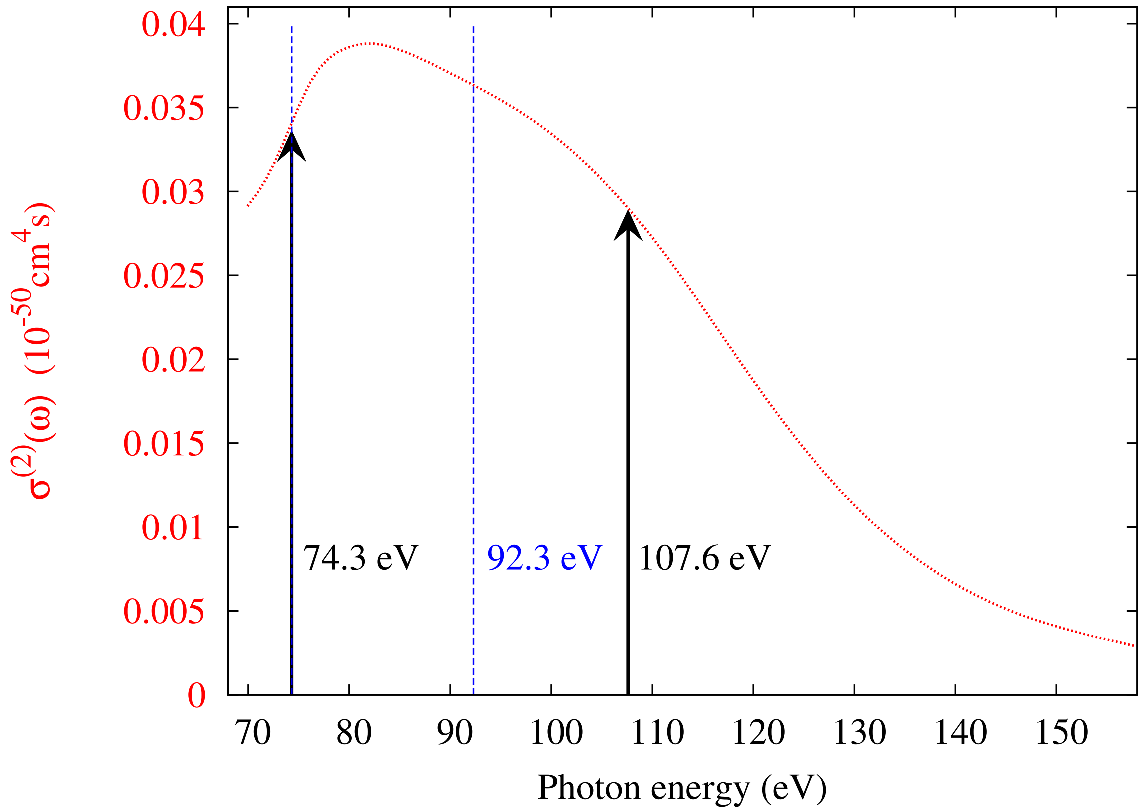

Supplementary Figure 1. The 2-photon absorption cross section $\sigma^{(2)}$ of xenon produced with the full model is shown in the range of the giant dipole resonance. The energy positions of the underlying resonances are indicated with black arrows for the TDCIS model (Y.-J. Chen, S. Pabst, A. Karamatskou, and R. Santra, manuscript in preparation), and with blue dashed lines for Wendin's results [1]. The first resonance energy is the same for both calculations, while the second energy differs considerably.

Supplementary Discussion

The surprising broadening of the 2-photon cross section curve with respect to the single-photon cross section is discussed in this section. Our theoretical analysis (i.e. the numerical solution of the Schrödinger equation) of the 2-photon cross section in the range of the $4d$ giant resonance within the full and the reduced model, respectively, shows a qualitative difference with respect to the corresponding 1-photon cross section curves.

The reduced model predicts a narrower 2-photon cross section curve compared to the 1-photon ionization cross section, whereas the full model 2-photon cross section curve is significantly broader than the 1-photon cross section curve and exhibits resonance-like features. In the following a simple perturbative model is applied to the results of both the reduced and the full model in order to clarify the surprising nature of the broadening in the 2-photon cross section of the full model.

If perturbation theory can be applied and the photon energy lies is the vicinity of a single, isolated 1-photon resonance (intermediate state), the 2-photon cross section can be factorized into two 1-photon cross sections. In general, the cross section for the transition between the initial bound state and the final continuum state is obtained as the modulus of the transition matrix element squared. In standard perturbation theory, where the interaction with the light field described by $\hat{H}_{\rm int}$ can be treated as a perturbation, the transition matrix element between the initial and final states for 2-photon absorption is given by:

$$\sum_{M_{\rm res}} \frac{\langle F|\hat{H}_{\rm int}|M_{\rm res}\rangle\langle M_{\rm res}|\hat{H}_{\rm int}|I\rangle}{E - E_{\rm res} + \frac{i}{2}\Gamma_{M_{\rm res}} + E_I}, \qquad \text{(Supplementary Equation 1)}$$

where $\Gamma_{M_{\rm res}}$ is the decay width of the resonance state $M_{\rm res}$, and E is the photon energy.

For a free-free transition the photoabsorption probability decreases monotonically with photon energy. Let us assume for the sake of simplicity that the transition matrix element from the intermediate state to the continuum follows (for not too small photon energy values) a simple $E^{-13/4}$ dependence: as Bethe and Salpeter show in Sec. 70 of their text book [2] the energy dependence $E^{-l-7/2}$ results for the cross section in the Born approximation (plane wave approximation without any screening effect). Therefore, for a resonance state exhibiting mostly f-character (angular momentum $l = 3$) the exponent becomes $-13/2$ for the cross section. This means that for a single, isolated intermediate state, the 2-photon cross section is obtained by multiplying the 1-photon ionization cross section by the energy-dependent factor. The result for the reduced model is shown in Fig. 4a) of the main text (blue curve). The 1-photon ionization cross section (black curve) multiplied by $1/E^{13/2}$ is shifted to smaller energies compared to the 1-photon curve and exhibits also the smaller width of the 2-photon cross section peak (red curve). However, in the full model this simple approximation breaks down. As shown in Fig. 4b) the resulting curve for the 2-photon absorption cross section within this simplified model (blue curve) is indeed shifted to a smaller energy; but the width is also decreased in strong contrast

to the calculated 2-photon cross section curve in the full model, which is broadened (red curve). Furthermore, the dashed blue curve underestimates the experimental cross section especially at 140 eV by a considerable factor. This demonstrates that this simple model does not capture the physics of the full model if only a single resonance is taken into account as the intermediate state.

Thus, the fact that the 2-photon cross section curve is significantly broader than the 1-photon cross section provides evidence for the concept of more than one resonance underlying the giant resonance [3, 4, 1]. In this case there is a sum over several resonance states in the cross section expression (Supplementary Equation 1) and interference terms between overlapping resonances arise, which can broaden the cross section curve. Moreover, the shape of the curve contains overlapping peak-like structures (Supplementary Fig. 1). The curve seems to accommodate two (or more) resonances. Indeed, it has been argued that the giant dipole resonance contains two underlying resonances [1], but the 1-photon cross section is not a sensitive observable for this fact. Clearly, the 2-photon cross section represents a more sensitive observable for testing this hypothesis of two (or more) resonances, because especially at 140 eV the experimental results are described by the full model which gives a much larger cross section. This led us to the conclusion that the curve must be broader than initially assumed. Motivated by these findings a characterization of the resonance energies was recently performed within TDCIS (Y.-J. Chen, S. Pabst, A. Karamatskou, and R. Santra, manuscript in preparation): The calculation reveals two underlying resonance states, whose real parts lie at 74.3 and 107.6 eV, respectively. The energies are marked by arrows in the Supplementary Fig. 1. These positions are consistent with the structure apparent in the 2-photon cross section curve. In the same figure we indicate for comparison by blue dashed lines the positions of the energy poles found by Wendin [1]. The positions of the first resonance energy coincide perfectly, while the second resonance energy within TDCIS is larger compared to Wendin's calculation. It must be emphasized that the two methods of calculation and the quantities used for obtaining the energy poles are very different. While Wendin uses a one-channel approximation (together with a random phase approximation with exchange) to evaluate the dielectric function matrix elements, we use the many-body wavefunction in the CIS approximation to extract the eigenstates of the system [5, 6]. Also, in our approach we do not include ground state correlations in contrast to Wendin who shows that taking

into account ground state correlations can result in a narrower cross section curve [1]. In principle, ground state correlations would not affect the spacing between the resonances but they could lead to different transition matrix elements. This could result in different interference behavior, which, in turn, could change the shape of the two-photon cross section. This question, which we cannot address, remains a challenging task and further experimental measurements and theoretical investigations are needed to determine the exact two-photon cross section. However, the agreement between theory and experiment is very good when including more than a single intermediate state, which leads to a broadening of the cross section.

In summary, the excellent agreement between the experimental results and our full model calculations of the 2-photon cross section (present work) as well as the resonance energy calculations (Y.-J. Chen, S. Pabst, A. Karamatskou, and R. Santra, manuscript in preparation) strongly indicate that the form and the broadening of the 2-photon cross section curve can be explained when taking into account the existence of more than one resonance, which are overlapping, and therefore interfering, in the range of the $4d$ giant resonance. Experimental measurement techniques in the nonlinear regime are sensitive to the influence of the underlying resonances of the giant resonance in xenon.

Supplementary References

[1] Wendin G., Collective effects in atomic photoabsorption spectra. III. Collective resonance in the $4d^{10}$ shell in Xe. *J. Phys. B: At. Mol. Phys.* **6**, 42 (1973)

[2] Bethe H. A. & Salpeter E. E., Quantum Mechanics of One- and Two- Electron Atoms, Dover ed. (Mineola, N.Y., 2008)

[3] Danos, M., & Greiner, W., Damping of the Giant Resonance in Heavy Nuclei. *Phys. Rev.* **138**, B876-B891 (1965)

[4] Veyssibre A., Beil H., Bergbre R., Carlos P., & Leprtre A., Photoneutron cross sections of ^{208}Pb and ^{197}Au. *Nucl. Phys. A* **159**, 561–576 (1970).

[5] Greenman, L., Ho, P. J., Pabst, S., Kamarchik, E., Mazziotti, D. A., & Santra, R., Implementation of the time-dependent configuration-interaction singles method for atomic strong-field processes. *Phys. Rev. A* **82**, 023406 (2010).

[6] Karamatskou, A., Pabst, S., and Santra, R., Adiabaticity and diabaticity in strong-field ionization. *Phys. Rev. A* **87**, 043422 (2013).
